# Microgeophysics and geomatics data integration reveals the internal fracturing conditions of the statue of Ramses II (Museo Egizio, Torino, Italy)

**DOI:** 10.1038/s41598-022-14300-z

**Published:** 2022-06-15

**Authors:** Chiara Colombero, P. Dabove, N. Grasso, F. Khosro Anjom, F. Pace, S. Aicardi

**Affiliations:** 1 Department of Environment, Land and Infrastructure Engineering, Politecnico Di Torino, Torino, Italy; 2 Fondazione Museo Delle Antichità Egizie Di Torino, Torino, Italy

**Keywords:** Geophysics, Engineering

## Abstract

The combined acquisition of 3D ultrasonic tomography and radar scans is growing for cultural heritage diagnostics. Both methods proved to be efficient in the detection and location of fractures and weaknesses within the investigated artefacts. Although the two techniques are widely applied together, an integrated approach for data interpretation is still missing. We present the results of radar and ultrasonic prospections carried out on the statue of the young Ramses II, an absolute masterpiece of the Egyptian art preserved in the collection of the Museo Egizio of Torino (Italy). Geophysical results are incorporated within the 3D model of the statue retrieved from total station measurements, ground-based and handheld laser scanning. A data integration approach is then proposed for the joint interpretation of the geophysical results, exploiting the final ultrasonic velocity model and radar attribute analysis (i.e. local dissimilarity computation) to define a combined damage index. The proposed methodology is efficient in fracture detection and location and improves the readability of the final results also for non-expert geophysical interpreters, offering guidance to the museum for preservation and restoration of the masterpiece.

## Introduction

Throughout history, ornamental stones have been the most common materials of monumental sculpture. As all rock types, stones can experience physico-chemical weathering, deterioration and damage during time^1^. The consequences may be even more serious if natural predisposing heterogeneities in the rock texture and microfractures are already present in the original materials^2^. Cultural heritage preservation implies therefore a detailed knowledge of the structural integrity and fracturing conditions of artefacts, monuments and historical buildings^3^. This characterization also plays a key role in identifying previous restoration activities and planning new ones. Dealing with artworks, the investigation method should be non-destructive and non-invasive and only imply a temporary contact between the prospecting instrumentation and the investigated object. Geophysical methods, originally intended for the investigation of the subsurface, can be applied for the detection of weaknesses and fractures within small-scale objects, thanks to the advances in both instrumentation size/portability and resolution of the results^4^. The application of geophysical methods for cultural heritage diagnostics and monitoring is also referred to as microgeophysics^5^ and represents a growing field of application. Between the available methods, ground-penetrating radar (GPR) and ultrasonic techniques have largely demonstrated their applicability for the detection of fractures and weaknesses in stone artworks. The two methods have been coupled in several studies for the screening of structural and ornamental elements in historical buildings^6–10^. In GPR data, fractures can show different signatures depending on their orientation, geometry and filling materials^11–13^, e.g. abrupt interruptions in the continuity of the reflection horizons, diffraction patterns, high-amplitude back reflections of the electromagnetic (EM) signal at the discontinuities. As largely applied in seismic reflection, GPR attribute analyses can support data interpretation and fracture recognition^14^. Between the large variety of attributes that can be computed on radar data, texture attributes have proven to provide clear images of the distribution and shape of potential targets^15,16^.

In GPR acquisitions, a regular surface is needed for the coupling of the antenna with the investigated object. On complex 3D geometries, typical of statues and other artworks, the 3D ultrasonic tomography is consequently often carried out alone^17,18^. In these conditions, tomographic acquisition and processing are also complicated by the need for accurate positioning of the ultrasonic sources and receivers. Geomatics can support the reconstruction of the survey geometry, by providing 3D detailed and measurable models of the artefacts, through image-based and/or range-based modeling coupled with total station measurements^19–24^. The obtained 3D models also allow for the restitution of the geophysical results in a more effective and communicative way.

When both GPR scans and ultrasonic tomography are carried out on the same object of investigation, common practice for data interpretation is the visual comparison between the anomalous reflection amplitude patterns in the radar sections and the location of low velocity anomalies in the ultrasonic results. An attempt towards real data integration was proposed by few authors^7^, displaying the ratio between the GPR amplitude of reflection and the ultrasonic velocity to highlight the areas with higher damage on a Roman marble slab. The most degraded areas were indeed characterized by the lowest velocities and the highest amplitudes of reflection. As a consequence, the computed ratio showed the highest values along the weaknesses. However, in presence of widespread fracturing, material heterogeneities and intricate object geometries, the combination of the results may become even more challenging due to complex wave propagation. Further attempts of data integration are therefore needed to exploit the combined use of the two geophysical techniques for cultural heritage screening and diagnostics. A new approach for GPR and ultrasonic data integration is proposed in this study for the statue of Ramses II, preserved in the collection of the Museo Egizio of Torino (Italy). Non-invasive diagnostic tools were needed to evaluate the internal conditions and integrity of the sculpture in view of a possible restoration.

## The statue of Ramses II and survey design

**Figure 1:**
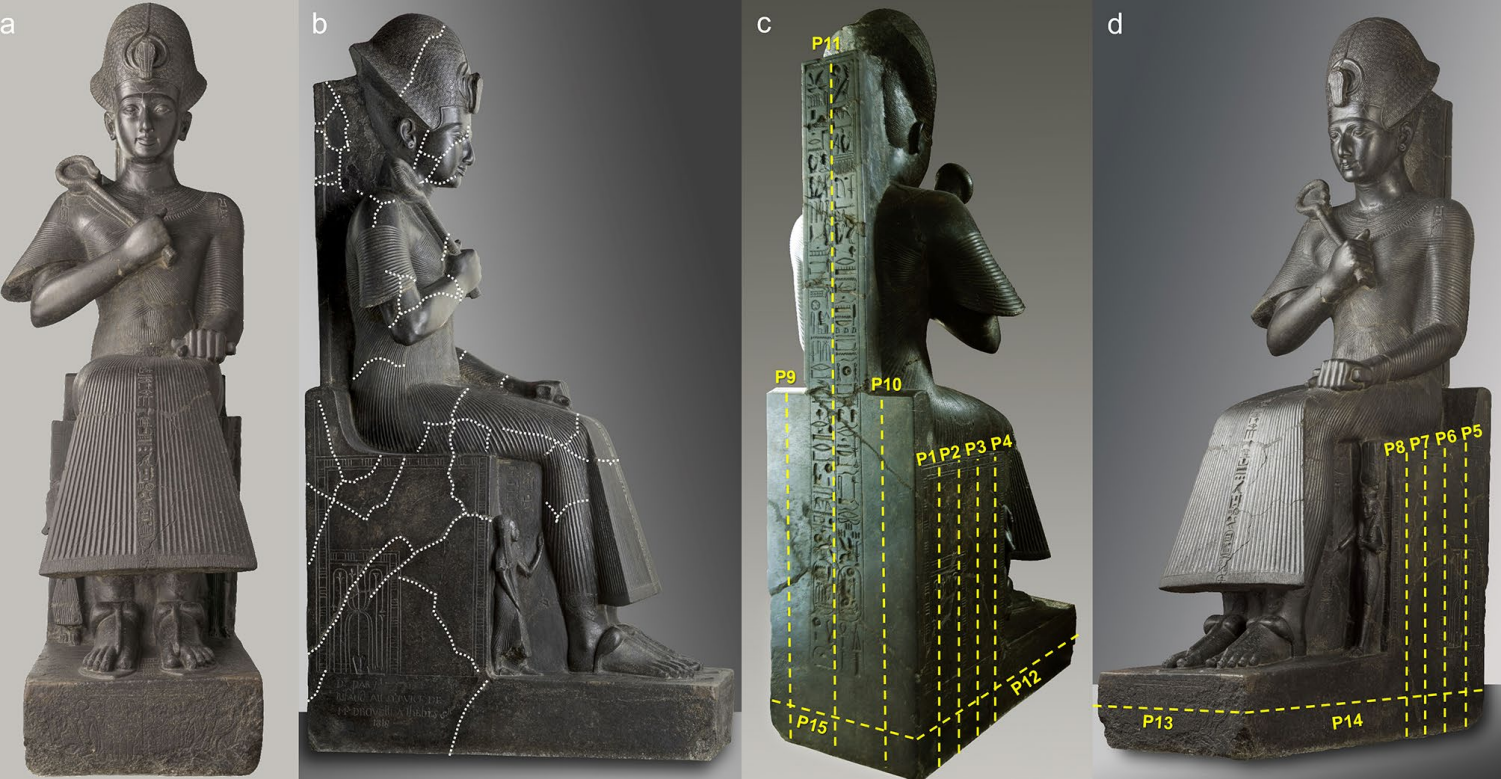
The statue of the young Ramses II (Museo Egizio of Torino): (**a**) frontal view; (**b**) view from the right side, with superficial fractures highlighted by white dotted lines; (**c**) back of the statue; (**d**) left side. GPR scan positions (P1 to P15) are marked by yellow dashed lines in (**c**) and (**d**). Pictures modified from the Museo Egizio online collection (https://collezioni.museoegizio.it/en-GB/material/Cat_1380/).

**Figure 2:**
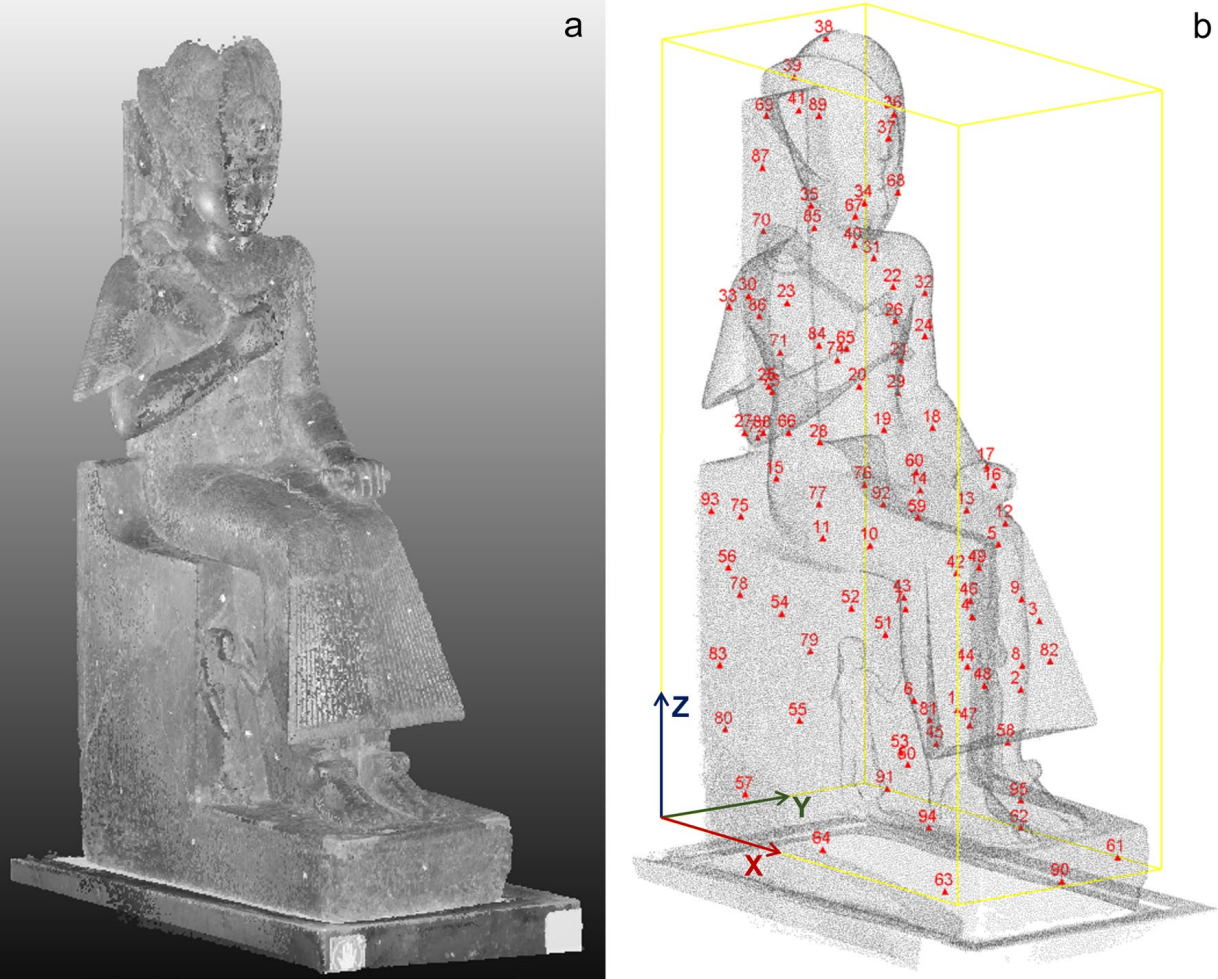
3D model of the statue of Ramses II. (**a**) Point cloud with RGB colors obtained with ground-based laser scanning. (**b**) Complete point cloud obtained through handheld and ground-based laser scanning, with indication of the 3D ultrasonic tomography source and receiver locations (red triangles, georeferenced through total station measurements) and of the local reference system adopted in the study. Figure created in CloudCompare (version 2.12.2, https://www.danielgm.net/cc/).

Within the sculptures of the Museo Egizio of Torino, the statue of the young Ramses II (Fig. 1) is considered an absolute masterpiece of the Egyptian art. The statue has dimensions of approximately 1.96 m (height), 0.47 m (width at the base) and 1.05 m (maximum depth at the base). It was carved in fine-grained diorite at the beginning of his reign (1279–1213 BC) and then exposed in a temple in Thebes. The pharaoh is enthroned, with his wife Nefertari and son Amonherkhepeshef represented as small statues on the sides of the legs. The statue was probably reassembled at the beginning of the XIX century and then restored. Visual inspection highlights indeed many superficial fractures, that are mostly located on the surfaces of the throne, on the back support, on the legs, arms and hat of the pharaoh (Fig. 1b). Combined 3D GPR scans and ultrasonic tomography were therefore acquired on the statue, to identify the inner persistence of the widespread fracture pattern present on the surface and to locate the main volumes of structural weakness. Fifteen radar scans were acquired on the vertical planar surfaces of the throne (P1 to P15 in Fig. 1c–d) with antenna in dual polarization (i.e. longitudinal or transverse to the scan direction), while 3D ultrasonic tomography covered the entire sculpture, with a distributed grid of 95 source and receiver positions (Fig. 2b). A 3D model of the statue was needed to reconstruct the geometry of the ultrasonic survey and accurately compute the source-receiver distances. The same model was used to place the radar scans in a 3D domain and to compare the geophysical results in overlapping sections of the statue. The small space behind the statue (0.28 m to the rear wall) and the poor lighting made it impossible to carry out classical photogrammetric techniques. The 3D model of the statue was therefore retrieved through the combination of total station measurements, ground-based and handheld laser scanning. The obtained 3D point cloud (Fig. 2) was further used to visualize the integrated geophysical results in an effective way.

A detail description of geomatics and geophysics data acquisition, processing and integration is provided in the “Methods” section.

## Radar scans

**Figure 3:**
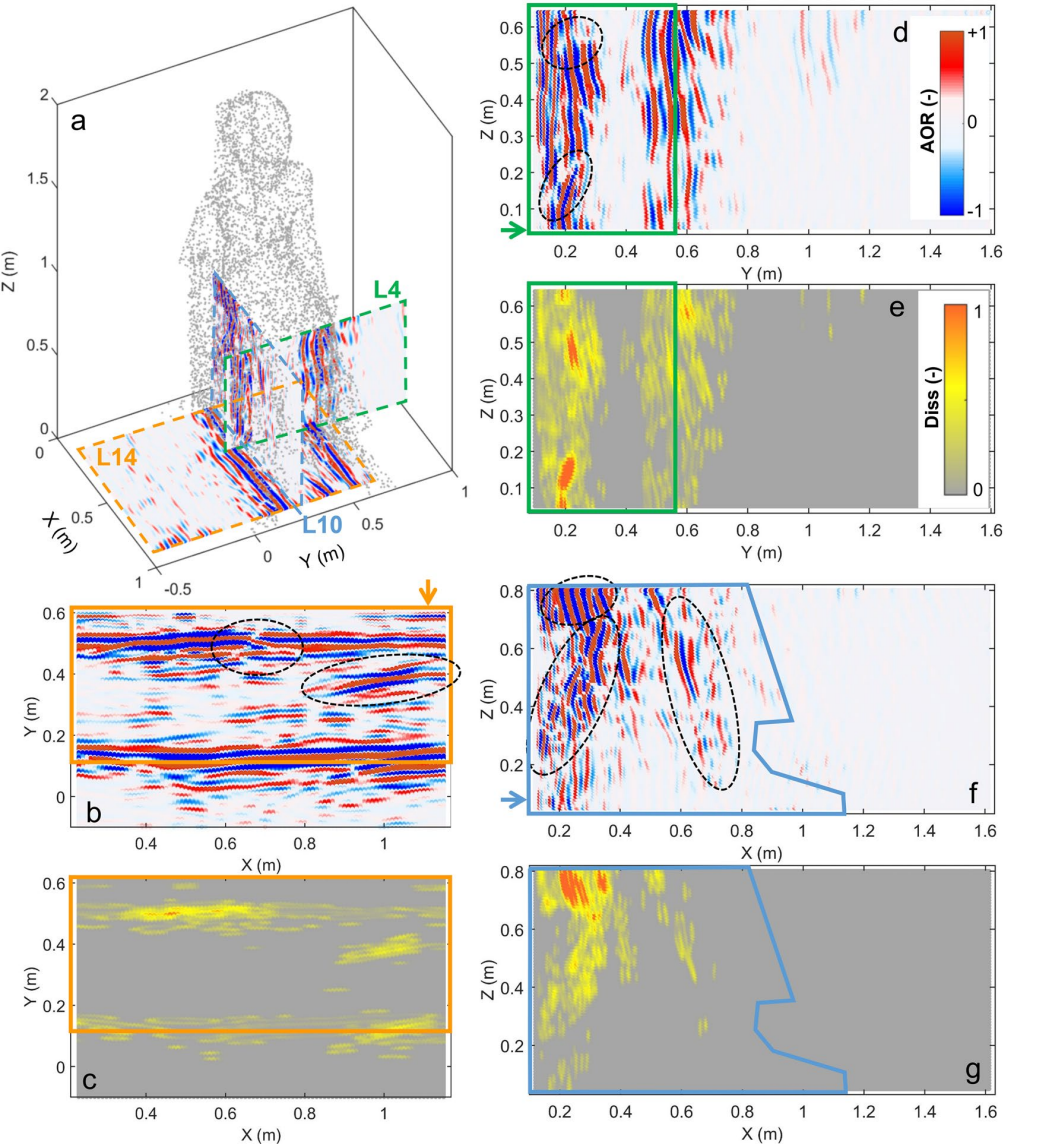
(**a**) Subsampled 3D point cloud of the statue with a selection of three orthogonal radar scans acquired in longitudinal polarization (L4, L10 and L14). (**b**–**c**) Scan L14: (**b**) processed radargram, (**c**) local dissimilarity. (**d**–**e**) Scan L4: (**d**) processed radargram, (**e**) local dissimilarity. (**f**–**g**) Scan L10: (**f**) processed radargram, (**g**) local dissimilarity. In (**b**) to (**g**) the GPR results inside the statue are bordered by colored polygons. The arrows indicate the side on which the GPR antenna was located during the acquisition. Figure created in Matlab (version R2021b, https://it.mathworks.com/products/matlab.html).

A selection of three perpendicular processed radargrams is reported in Fig. 3 (P4, P10 and P14 in Fig. 1c–d). These sections were acquired with the radar antenna having longitudinal (L) polarization. For each section (L4, L10, L14), the amplitude of reflection (AOR in Fig. 3b,d,f) is shown in comparison with the local dissimilarity distribution (Fig. 3c,e,g). The base of the statue (L14, orange polygon in Fig. 3b) appears quite transparent to the penetration of the EM signal, with a clear continuous reflection at the opposite side of the base, caused by the contrast in EM properties between the stone material and the air outside the statue. Nevertheless, some abrupt interruptions in the continuity of the near horizontal reflections and low dipping reflections with high amplitude are visible in the processed radargrams (circled in Fig. 3b). These anomalous areas are well identified by the highest local dissimilarity values (Fig. 3c). Reflection patterns on the lateral sides of the throne are even more intricate (Fig. 3d,f). The penetration depth from the back of the statue (i.e. along the X-axis) is insufficient to image the EM contrast between the stone material of the feet, legs and dress and the air in front of the statue (see Fig. 3f,g). Strong signal attenuation, scattering and back-reflections (e.g., around X = 0.6–0.7 m, Fig. 3f) are likely the cause of this reduced penetration. Complex reflection patterns with high amplitude and linear cross-cutting interruptions are observed in all the sections and their location is well represented by the local dissimilarity anomalies (Fig. 3e,g).

**Figure 4:**
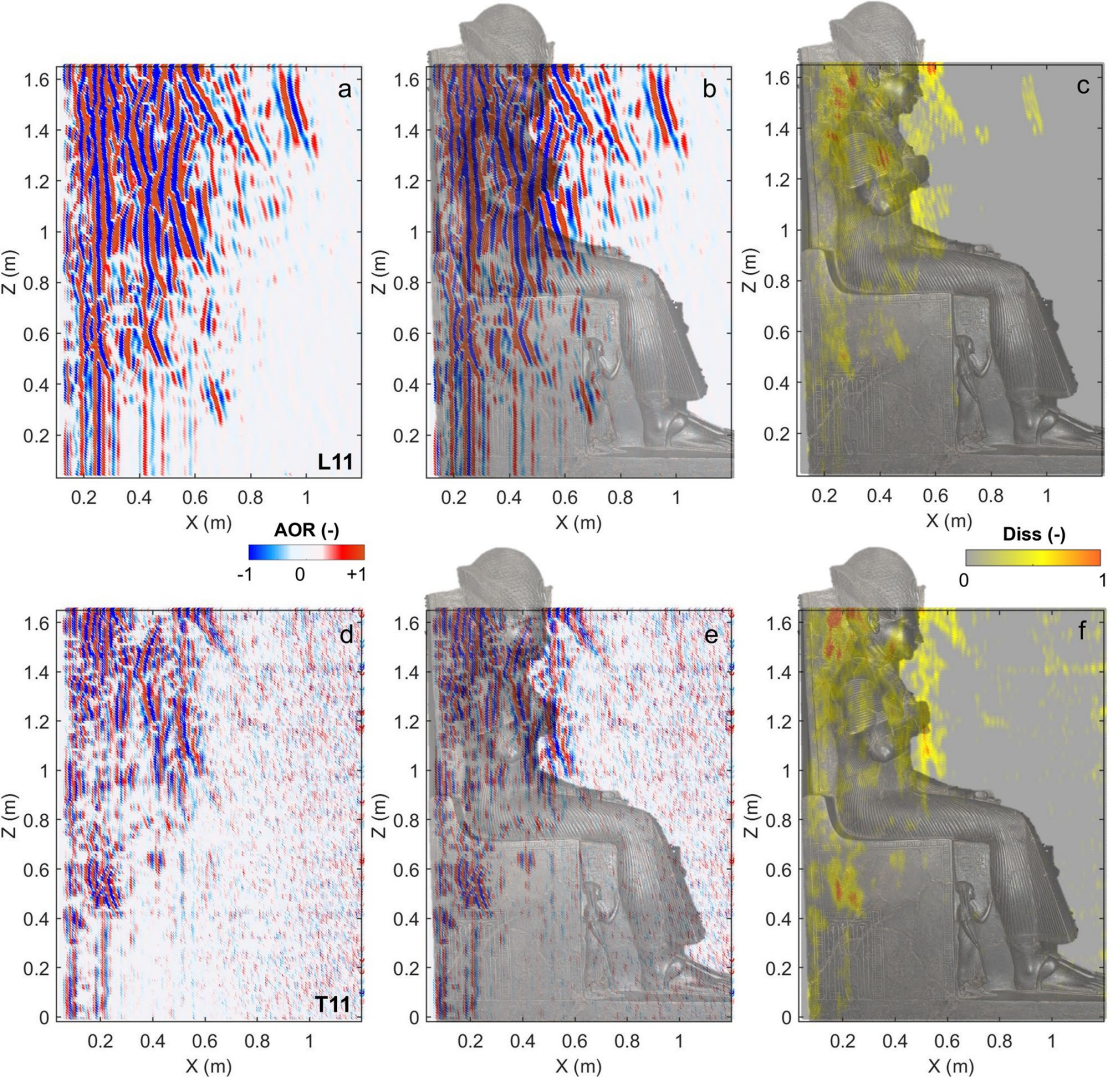
Comparison of GPR results obtained in dual polarization along the radar scan P11. (**a**–**c**) Longitudinal polarization (L11): (**a**) processed radargram, (**b**) processed radargram with the overlapped image of the statue, (**c**) local dissimilarity. (**d**–**f**) Transverse polarization (T11): (**d**) processed radargram, (**e**) processed radargram with the overlapped image of the statue, (**f**) local dissimilarity. Figure created in Matlab (version R2021b, https://it.mathworks.com/products/matlab.html).

A comparison between the longitudinal (L) and transverse (T) polarization results is shown in Fig. 4 (L11 vs T11) for the longest radar scan, acquired on the back of the statue (P11 in Fig. 1c).

Both polarizations display similar reflection patterns and features, even if longitudinal results (L11, Fig. 4a) show less scattering and attenuation of the signal, with higher penetration depth with respect to the transverse radargram (T11, Fig. 4d). The bottom of the throne is found to be quite transparent to the EM signal penetration in both polarizations (Fig. 4b,e). On the top of the statue (at Z > 0.8 m), the strongest reflections can be referred to the visible elements or edges of the statue, at the transition between the stone material and the outside. In particular, the throne edges and other point-like elements, such as the arms, right hand and head originate marked reflections in the radargrams, with 3D superimposition of the effects. Despite these geometric effects, the amplitude of the reflection is not found to be homogeneous within the statue. Anomalous reflection patterns are observable in the upper part of the throne, above the hips and in the face of Ramses II. They are also particularly widespread in the back support of the statue, close to the head of the statue (Fig. 4b,e). The local dissimilarity anomalies of the two polarizations show consistent locations and clearly highlight the most fractured areas (Fig. 4c,f).

## 3D ultrasonic tomography

**Figure 5:**
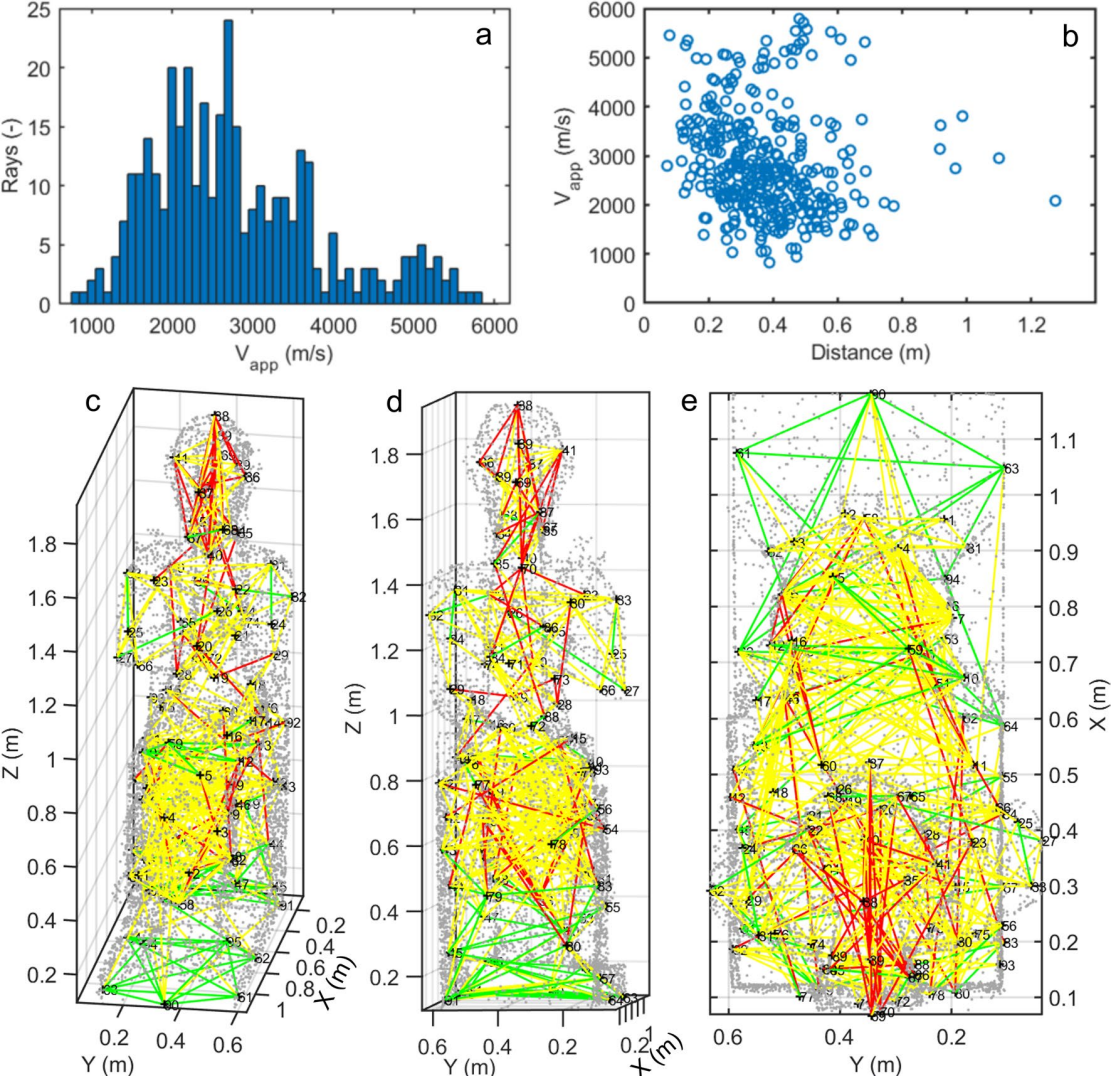
Apparent velocities obtained from the 3D ultrasonic measurements. (**a**) Histogram of the apparent velocity distribution (Vapp). (**b**) Apparent velocity values plotted as a function of the Euclidean source-receiver distance of the measured travel paths. (**c**–**e**) Measured paths with the classification of the apparent velocities in three classes: (**c**) frontal view, (**d**) view from the back, (**e**) view from the top. Figure created in Matlab (version R2021b, https://it.mathworks.com/products/matlab.html).

Given the irregular grid of 95 measure points (Fig. 2b), a total of 356 ultrasonic source-receiver pairs, which line-of-sight was entirely contained in the volume of the statue, were investigated with ultrasonic measurements. The computed apparent velocities (i.e. obtained as the ratio between the Euclidean source-receiver distance and the picked travel time) are reported in Fig. 5. The majority of the measured paths showed apparent velocities in the range between 1500 m/s and 3700 m/s (Fig. 5a). The average value is 2814 m/s (V_app,m_), with standard deviation equal to 1056 m/s (V_app,std_), indicating a large variability in the obtained results. Considering velocities higher than 4500–5000 m/s as representative for the intact rock type, only less than a tenth of the acquired measurements showed velocities values in this range. The comparison between source-receiver distances and the measured apparent velocities (Fig. 5b) does not show a bias in the distribution. The absence of a clear correlation between the measured distances and the estimated velocities indicates a good selection of the investigated travel paths and reflects the overall high degree of weakness of the statue. The apparent velocities are plotted in Fig. 5c–e along the measured travel paths and classified in three ranges: values higher than 3870 m/s (V_app,m_ + V_app,std_) in green, values lower than 1758 m/s (V_app,m_- V_app,std_) in red, and the intermediate values in yellow. The rays showing the highest apparent velocities are located predominantly in the base and lower part of the throne. A few rays belonging to the same class are also depicted in the upper part of the legs and, locally, on the arms of Ramses II. The lowest apparent velocities are positioned in the head, bust and upper part of the throne.

**Figure 6:**
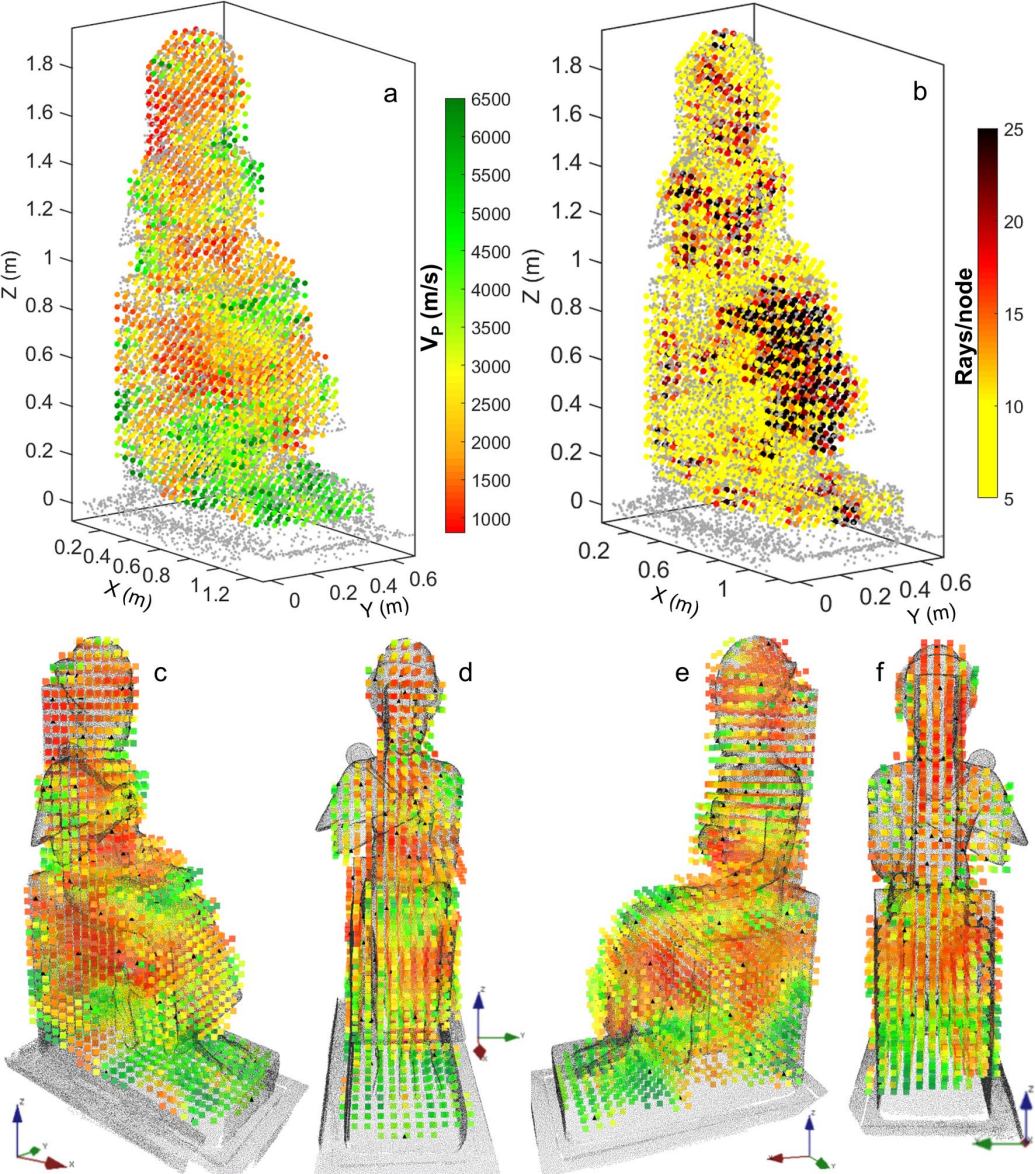
Results of the 3D ultrasonic tomography inversion. (**a**) Final velocity model and (**b**) ray coverage visualized in the simplified point cloud of the statue (Matlab R2021b, https://it.mathworks.com/products/matlab.html). (**c**–**f**) Final velocity model from different views: (**c**) right frontal side, (**d**) front, (**e**) left frontal side, (**f**) back (point cloud rendering in Voxler, Demo version 4.6.913, https://www.goldensoftware.com/products/voxler).

These preliminary considerations on the apparent velocities are confirmed by the results of traveltime tomographic inversion (Fig. 6). The final velocity model (Fig. 6a), obtained at the 30th iteration, has a root-mean-square deviation between measured and calculated traveltimes lower than 5%. The cells without ray coverage (Fig. 6b) are excluded from the visualization. Low velocity anomalies, likely related to the presence of deep fractures and/or reassembled materials, are found in the upper part of the throne, above the hips, in the head and back support of Ramses II.

## Data integration

**Figure 7:**
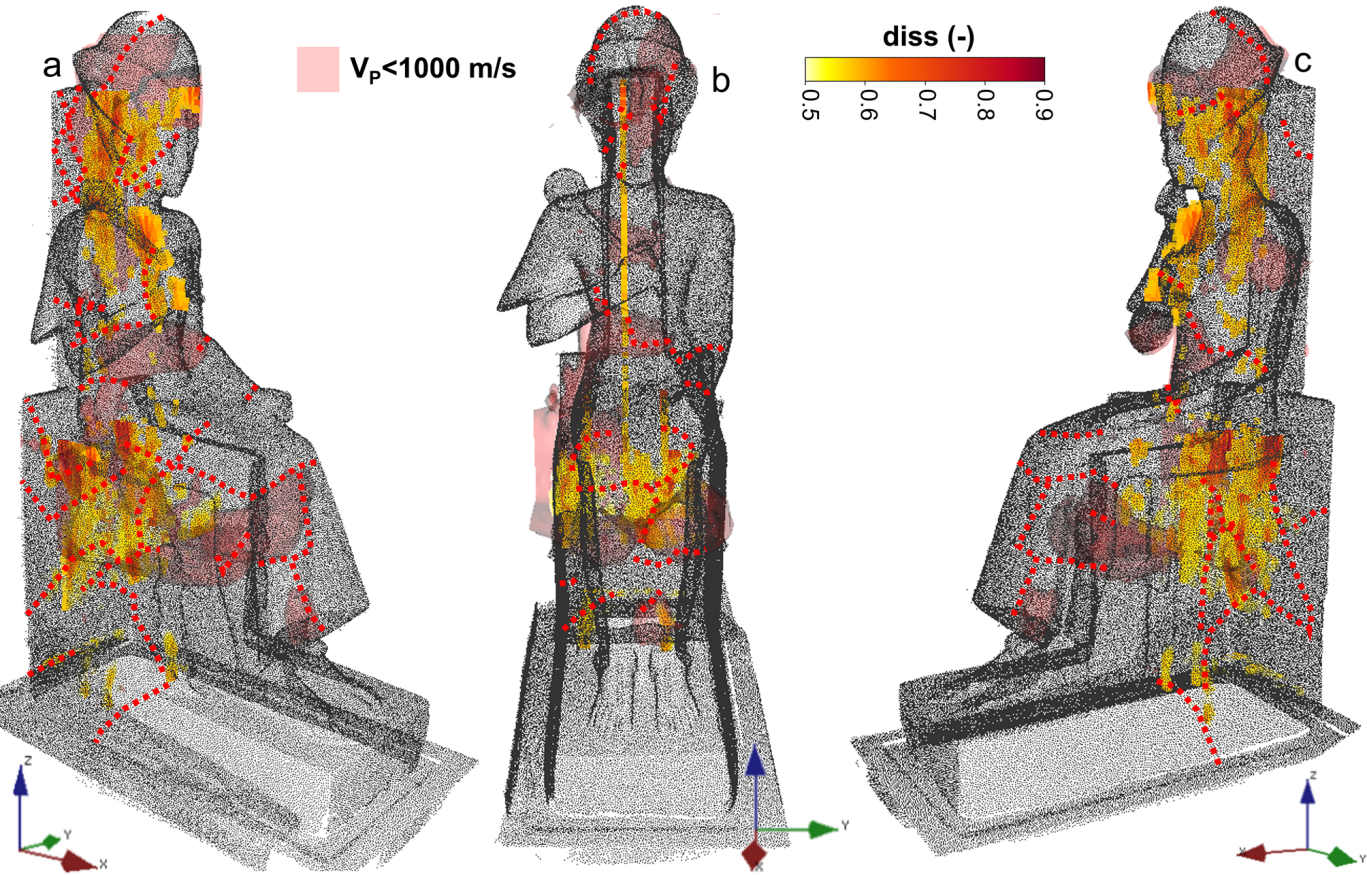
3D comparison between local dissimilarity anomalies and isosurfaces with ultrasonic velocity lower than 1000 m/s: (**a**) right side, (**b**) front view, (**c**) left side of the statue. The main superficial fracture traces are highlighted by red dotted lines. Figure created in Voxler (Demo version 4.6.913, https://www.goldensoftware.com/products/voxler).

Both GPR scans and ultrasonic tomography were proved to be highly suitable for the detection of fractures and weaknesses within the statue. The 3D ultrasonic tomography enabled the investigation of the entire volume of the statue while GPR scans were limited to the available planar surfaces of the statue. The upper part of the head was therefore inspected by only the ultrasonic measurements. Similarly, due to the limitations of the penetration depth of the EM signals, likely linked to scattering, back-reflections and strong attenuation at the inner fractures, the frontal parts of the dress and legs of Ramses II were not fully characterized by the GPR scans. Despite the different acquisition geometries of the two surveys, the location of both low velocity and radar anomalies related to fracturing is consistent. Figure 7 shows a comparison between the areas characterized by the highest local dissimilarity values (> 0.5) in the GPR sections and the isosurfaces of ultrasonic velocity lower than 1000 m/s. The local dissimilarity is shown for all the results in longitudinal polarization. GPR scans with longitudinal and transverse polarizations disclosed indeed similar features (Fig. 4), but with higher amplitude and penetration depth in the longitudinal configuration. The presence of major pervasive and deep fractures in the upper part of the throne, in the head and waist of Ramses II, is definitively confirmed.

**Figure 8:**
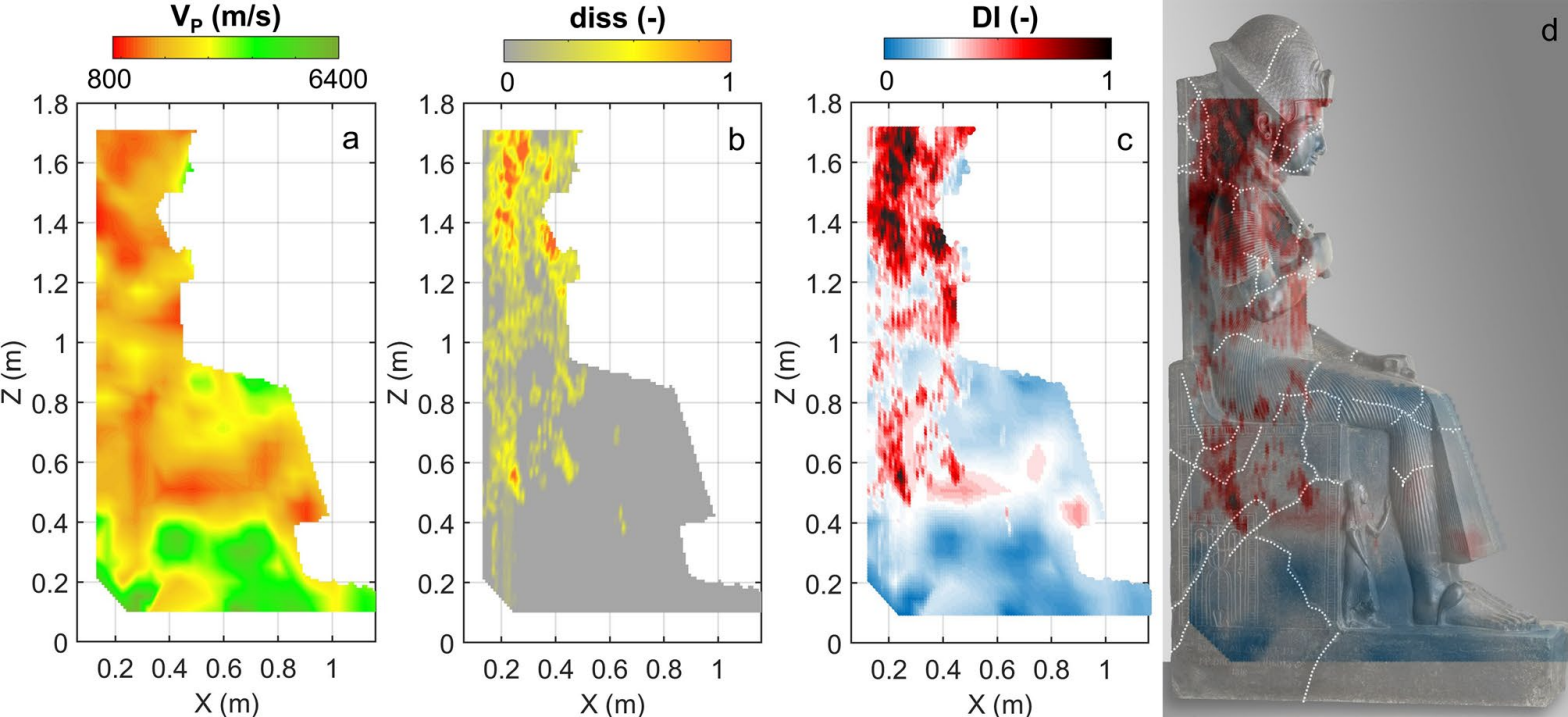
(**a**) Ultrasonic velocity, (**b**) local dissimilarity (L11 scan), (**c**–**d**) damage index on the axial vertical section of the statue of Ramses II. In (**d**) superficial fracture traces are highlighted on the image of the statue by white dashed lines. Figure created in Matlab (version R2021b, https://it.mathworks.com/products/matlab.html).

For a more comprehensive data integration, the radar and ultrasonic results obtained along common sections were further compared. An example is shown in Fig. 8 for the most representative vertical axial section of the statue investigated by both methods. The values of ultrasonic velocity (Fig. 8a) and of local dissimilarity (Fig. 8b) were resampled and interpolated to common grid points along the section. We then defined a novel and simple damage index (DI, Fig. 8d) to combine the results, following:

1 $$\mathrm{DI}=\frac{1}{2}\left[\left(1-\frac{{\mathrm{V}}_{\mathrm{P}}}{{\mathrm{V}}_{\mathrm{P},\mathrm{max}} }\right)+\mathrm{diss}\right],$$

where the ultrasonic velocity V_P_ of each grid point is normalized to the maximum velocity of the final velocity model (V_P,max_).

In massive and compact portions of the statue, the velocity is expected to be high (close to V_P,max_) while the local dissimilarity values should be low. The DI will therefore be close to 0. In the fractured portions of the statue, the DI will conversely tend to 1. The visualization of the combined DI (Fig. 8c) facilitates the identification of the major weakness in the investigated section if compared to the results of the single methods (Fig. 8a and b). The location of the superficial fracture traces on the right side of the throne and the obtained DI are compared in Fig. 8d. The fractures located in the head, shoulders and back support of the statue are associated with the highest DI values. They can therefore be interpreted as persistent at depth and represent the major weaknesses of the statue. The fracture traces visible on the upper part of the throne and along the waist of Ramses II show similar signatures.

## Conclusions

3D radar scans and ultrasonic tomography were applied to assess the integrity of the statue of the young Ramses II. The reconstruction of a 3D model of the statue enabled/ensured an accurate processing of the ultrasonic measurements and hence improved the interpretation of all the geophysical results. While the recognition of low velocity anomalies from the ultrasonic results is straightforward, the interpretation of complex reflection patterns in the radar scans may be challenging. Textural attributes, and particularly local dissimilarity computation, supported the identification of the anomalous areas in the radar section, thus precisely constraining the fracture locations. We proposed an effective approach for ultrasonic and radar data integration, based on the computation of a Damage Index accounting for both geophysical results. This novel index is effective in locating the fracture presence and facilitates the interpretation of the geophysical results also by non-expert geophysical interpreters, offering guidance to the Museo Egizio for cultural heritage preservation. The integrated results highlighted indeed the presence of major persistent weaknesses within the statue, thus supporting the hypothesis of a past reassembly and reconstruction of the sculpture. The outcomes of this study also offer solid bases for the design of potential future restoration activities on the masterpiece.

## Methods

### 3D model of the statue

The accurate positioning and interpretation of the geophysical prospections required a 3D model of the statue in a local reference system. In particular, the 3D ultrasonic source and receiver geometry was mandatory to reliably compute source-receiver distances and process the data with a tomographic approach. These 95 positions were marked on the statue by numbered stickers (diameter = 0.01 m). We used a Leica Nova MS50 total station and a mini prism to register the coordinates of their centers, with a maximum root mean square of 3 mm. The results were considered acceptable considering the area of the ultrasonic transducers to be applied at each position (diameter = 5 mm). To guarantee high performance in terms of precision and accuracy, a network of observations with high redundancy (4 view points) was created.

We then considered a combination of two geomatics techniques to fully reconstruct the 3D geometry of the statue. First, we scanned the statue using FARO S 350 HDR terrestrial laser scanner, which provides high-resolution images also in environments with poor lighting. Four scans with approximately 50% overlap were obtained, two from the front and two from the sides of the statue. The angular resolution was set to obtain a point approximately every 3.5 mm at 5-m distance. Given the close distance of the statue to the rear wall (0.28 m), the back of the statue remained unscanned. As a solution, we used the F6 SR handheld laser scanner (Stonex) that provides both high accuracy, even when the scanning is performed from a close proximity (0.25 cm minimum), and photographic texture of the object thanks to the RGB camera^25^. The acquisition was managed on the Mantis Vision Echo software (version 2.4.0, https://mantis-vision.com/professional-solutions/echo-software-about), to customize the acquisition parameters (e.g., camera and IR settings, range distance) and visualize the ongoing point cloud acquisition. The F6 SR is based on an emitter projecting a near infrared (NIR) light through Mantis Vision’s proprietary pattern onto the scene while a receiver calculates the distance of each mapped point through triangulation algorithm and of the 3D scene by stereoscopic parallax^26^.

The scans from the FARO instrument were manually processed in FARO Scene software (version 2019.2, https://www.faro.com/en/Products/Software/SCENE-Software). After a manual filtering operation, a three-dimensional model of the statue of Ramesses II consisting of about 4 million points was obtained. The scans from the F6 SR scanner were processed in Mantis Vision Echo software, where the data quality was verified and the blurred frames were removed. Both point clouds were georeferenced in the local coordinate system of total station measurements, with a standard deviation on the points of known coordinates of less than 5 mm. A cloud-to-cloud comparison using the open-source CloudCompare (version 2.12.2, https://www.danielgm.net/cc/) algorithm calculated the Euclidean distance between the two point clouds in 3 mm of mean difference, with a standard deviation of 2 mm. The results were considered acceptable for the purpose of geophysical data integration and interpretation. Possible location errors are close to the GPR data resolution and did not affected the computed ultrasonic velocities, for which source and receiver positions were previously retrieved by total station measurements. After merging the two point clouds and removing residual noise through the Statistical Outlier Removal (SOR) filtering algorithm, the final 3D model consisted of 185 million points with an average density per distance of 1 point/0.02 mm.

### Radar Scans

The scans were carried out with an IDS K2 radar connected to a 2-GHz IDS Aladdin antenna with dual polarization (i.e. parallel and perpendicular to the direction of acquisition). This survey configuration potentially allows identification of fractures with different orientations within the statue. In particular, we acquired four vertical parallel profiles at 10-cm spacing on the left (P1 to P4) and right (P5 to P8) side of the throne, three vertical parallel profiles at 12-cm spacing on the back of the statue (P9 to P11) and four horizontal profiles on the sides of the basement (P12 to P15). The vertical profiles were all acquired from the bottom to the top; the horizontal profiles were recorded from left to right on each side of the basement. The antenna footprint is 12 cm × 12 cm. For this reason, the spacing between the profiles ensured to scan all the available planar space around the statue. Due to the antenna footprint, a scan distance equal to approximately 6 cm was lost at the beginning and end of each profile. The surveyed lines were marked with paper tape on the statue. The distances between the profile ends and the origin of the local coordinate system adopted for all surveys were measured to ensure 3D data integration. A wheel encoder was further used to track the position of the antenna along the profiles. The recorded time along each scan was set to 30 ns, to ensure a sufficient penetration also in the widest volumes of the statue (e.g. from the front to the back of the basement). A total of 2048 samples were acquired on each trace (sampling frequency of around 68 GHz). The average distance between to subsequent traces along each scan is 0.01 m.

Raw radargrams were processed in Reflexw (version 9.1.3, https://www.sandmeier-geo.de/reflexw.html) with a standard processing sequence including (i) start time shift (to remove the initial signal delay and retrieve correct traveltimes in the statue); (ii) dewow (i.e. high-pass filtering to remove low-frequency electronic noise); (iii) background removal (i.e. average trace subtraction to attenuate horizontal striping); (iv) divergence compensation, to recover amplitude at depth. The velocity of propagation within the statue was computed exploiting a known distance (e.g. 0.47 m, from the left to right side of the basement) and the measured travel time (twt = 8.7 ns). A constant velocity of 0.11 m/ns was consequently used for time-to-depth conversion. Considering the central frequency of the antenna, the propagating wavelengths inside the statue are equal to 0.05 m. The vertical resolution, theoretically equal to one fourth of the wavelength, can therefore be conservatory considered close to 0.02 m.

Between the large variety of algorithms for attribute computation, we exploited the built-in textural attribute module of Reflexw. Texture refers to the fact that the radargram is discretized in a number of regular cells and the amplitude of reflection in each cell is discretized in *n* gray-levels. 2D gray-level co-occurrence matrices (GLCM) are then used to depict spatial relations between neighboring cells. In detail, a square GLCM is built for each cell (size = *n* x *n*). Each element in the matrix contains the occurrence frequency of the *n* gray level in the surroundings of the cell. The GLCM matrix is then normalized in order to obtain, at each position (i.e., row *i*, column *j*), the probability of occurrence *P*_*i,j*_ of a specific gray-level pattern. *P*_*i,j*_ is then used in various equations to compute the desired textural attribute (e.g., uniformity, local homogeneity, local dissimilarity, entropy). 12 gray levels were adopted for amplitude discretization (*n* = 12) and cells of size equal to 3 traces and 12 samples were chosen to maintain a centimetric resolution. Testing all the available built-in attributes, local dissimilarity, defined as:

2 $$Diss=\sum_{i=1}^{n}\sum_{j=1}^{n}\left|i-j\right|{P}_{i,j },$$

was found to be the optimal attribute to highlight contrasts and local amounts of amplitude variations likely due to fracture presence. Processed radargrams and local dissimilarity sections were then assembled in their 3D spatial configuration adopting the local reference system of the 3D model through an on-purpose developed Matlab (version R2021b, https://it.mathworks.com/products/matlab.html) code.

### 3D ultrasonic tomography

Ultrasonic measurements are carried out between a transmitter (Tx) and receiver (Rx) probe coupled with the investigated object. An ultrasonic pulse is send by Tx and its arrival at Rx is recorded. The travel time between Tx and Rx can be easily recognized and picked as the first arrival time on the recorded ultrasonic trace. An ultrasonic pulse generator (Pundit) connected to two 54-kHz probes was used. The Tx-Rx probes have an exponential shape, to minimize the contact surface with the statue (< 0.2 cm^2^). A 7-dB fixed gain amplifier and a variable gain amplifier (1–3–10 dB) were used to amplify the ultrasonic pulse along the longest travel paths. The ultrasonic waveforms were sampled at 50 MHz by a LeCroy Wave Jet oscilloscope. A trace average equal to 16 was set before data storage to improve the signal-to-noise ratio.

Source and receiver positions were identified with 95 numbered stickers, located at an average distance of 15 cm on all the available surfaces of the statue and georeferenced through total station measurements. Their location was chosen to maximize ray coverage within the statue and minimize uninvestigated volumes. The system delay introduced by the Tx-Rx probes was measured in 20.7 μs before starting the measurements. This delay was then subtracted to all the picked traveltimes. A total of 356 Tx-Rx pairs, which line-of-sight was entirely contained in the volume of the statue, were then identified and measured. First arrival times were manually picked through an on-purpose designed Matlab code. After correction for the probe delay, traveltimes were inverted using GeoTomCG software (version 4.0, https://geotom.net/), which performs inversions with the simultaneous iterative reconstruction technique (SIRT^27,28^). SIRT calculations modify an initial velocity model by repeated cycles in three steps: forward computation of model traveltimes, calculation of residuals and application of velocity corrections. Curved ray tracing performed with a revised form of ray bending^29^ is used in the software.

Using Tx-Rx coordinates (X, Y, Z), we built an initial model with cubic cells (dx = dy = dz = 0.05 m) and homogeneous velocity (average apparent velocity = 2800 m/s) and ran 30 inversion iterations to obtain the final velocity model. Considering the average apparent velocity and the central frequency of the signals, cell size was chosen equal to the average propagating wavelengths (0.05 m). All the cells of the model having no ray coverage where rejected.

## Data Availability

Data acquired in this study are available on request by contacting the corresponding author.
